# Comment on Dr. Stillman’s Paper

**Published:** 1918-04

**Authors:** A. W. Thornton


### COMMENT ON DR. STILLMAN'S PAPER

BY DR. A. W. THORNTON

*Attaching bridgework to natural abutments.*—Nature has established a very sound principle in regard to the stress we may expect a natural root to bear. For example, the anterior teeth which are used for incision, have only one root, but when we examine a molar tooth we find that the forces exerted upon its surfaces come from different directions according to the inclination of its plane surfaces; the total area is greater than that of the anterior teeth, and as a result, the tooth is provided with much stronger anchorage in the maxilla or mandible by having two or three, or sometimes more roots. It must logically follow then, that when the roots of two or more natural teeth are forced to carry not only the stress of mastication which would ordinarily come upon their own crowns, but also the stress which is placed upon the artificial restorations which are made to substitute for several more teeth that have been lost, then the life of those natural teeth used as abutments must be shortened in proportion to the excess of work they are are obliged to do. I do not suppose that any fixed law can ever be laid down to govern the amount of additional work that any root will do. That is something which must be left to the judgment of the operator, and he alone must decide for each individual case that is presented to him, what method of restoring the lost tissue will be most beneficial to his patient. I do not believe Dr. Stillman intended in his paper that the profession should return to the old method of inserting fourteen-tooth fixed bridges on four abutments, and yet that thought may be taken from his statement. I should be glad to hear Dr. Stillman enlarge upon that idea so that we may not misconstrue his meaning.

Dr. Stillman has emphasized, and rightly, too, the use of the anatomical articulator in the making of crowns and bridges. This is easy enough when extraction has been recently done and the teeth remaining are in their normal relation. We all know, however, that a great many of the cases we have to deal with are cases of long standing, and in many instances the teeth of the opposing arch have exfoliated to such a marked degree that anatomical articulation is impossible without excessive grinding of natural teeth that are otherwise sound. Sufficient grinding of these natural teeth usually involves devitalization or later pathologic pulp disturbances. Failure to grind, ruins either the esthetic harmony of outline or the anatomical articulation with its disastrous traumatic effects that have been pointed out to us this evening. I presume this is another problem which must be solved by the dentist as each case is brought under his care.

